# Dental implant failure and bone augmentation: A retrospective study

**DOI:** 10.4317/jced.60171

**Published:** 2023-03-01

**Authors:** Georgios S. Chatzopoulos, Larry F. Wolff

**Affiliations:** 1 Department of Developmental and Surgical Sciences, Division of Periodontology, School of Dentistry, University of Minnesota, 515 Delaware Street SE, Minneapolis, MN,55455, USA; 2 Department of Preventive Dentistry, Periodontology and Implant Biology, School of Dentistry, Aristotle University of Thessaloniki, 54124 Thessaloniki, Greece

## Abstract

**Background:**

To retrospectively assess the failure rate of implants placed in augmented and non-augmented sites and to investigate whether the time of implant and bone placement are associated with the risk of implant failure in a university setting.

**Material and Methods:**

In this retrospective study, data were retrieved from the electronic patient database of the University of Minnesota School of Dentistry, USA to identify patients older than 18 years of age who received dental implant treatment. Patient characteristics and the adequacy of available bone were retrieved from the patients’ dental records and analyzed. Performing sinus lift and/or alveolar ridge augmentation in stages or simultaneously with implant placement and the need for multiple bone regeneration procedures were recorded. Kaplan-Meier plots and Cox regression models were created to analyze the data.

**Results:**

Data from 553 implants were analyzed in the study. More than half of the implants were placed in the maxilla (56.8%) and posterior regions (74.3%). The overall survival rate was 96.9%. Sinus augmentation was performed in 19.5% of the cases, while in 12.1% of the included treatments an implant was placed simultaneously. Staged and simultaneous ridge augmentation occurred in 45.2% and 18.8% of the cases, respectively. Implants placed in an area following (*p*=0.018) or simultaneously (*p*=0.025) with sinus augmentation showed a significantly reduced survival. Cox regression analysis showed that smoking and simultaneous ridge augmentation and implant placement increased failure rates.

**Conclusions:**

Within the limitations of this study, implants placed in tobacco users as well as in augmented maxillary sinuses, simultaneously or in stages, and in augmented ridges lead to higher implant failure rates.

** Key words:**Bone grafting, dental implant, osseointegration, risk factor, survival rate, treatment outcome.

## Introduction

Dental implant-supported prostheses are commonly and successfully used in daily clinical practice to replace missing teeth with predictable long-term treatment outcomes (1). Presence of sufficient bone volume, both in width and height, is crucial for proper implant placement and osseointegration. In addition, it allows per-implant hard and soft tissue stability. Insufficient bone volume may lead to compromised soft tissue and esthetically unpleasing outcomes (2). Lack of bone volume is generally associated with alveolar ridge resorption and/or maxillary sinus pneumatization. Thus, bone augmentation procedures may be necessary to allow optimal implant placement that will lead to a long-term functional and esthetic outcome. Bone regeneration procedures may be performed simultaneously with the implant placement or separately (3,4).

Various augmentation techniques can be performed to preserve or reconstruct a resorbed alveolar ridge or pneumatized maxillary sinus. Ridge preservation is a surgical procedure that is performed at the time of tooth extraction or shortly later that aims to minimize the ridge resorption that occurs physiologically after a tooth extraction, while maximizing the bone formation within the extraction socket (5). Although this technique is valuable and advantageous, bone remodeling of the ridge and the buccal bone will still occur following tooth removal (6). Ridge augmentation after tooth extraction is frequently performed following the principles of guided bone regeneration which includes the use of bone grafting materials and barrier membranes (7). Alternatively, atrophic alveolar crests may be reconstructed by ridge split procedure or distraction osteogenesis techniques (8,9).

Maxillary sinus floor augmentation techniques have been developed to reconstruct pneumatized maxillary sinuses using grafting materials (10). Based on the residual bone height, the elevation of the sinus membrane can either be performed through a lateral window or through the crest (10,11). All augmentation procedures may be implemented prior to or at the same time with implant placement when the implant can achieve a sufficient primary stability. With respect to grafting materials, autogenous bone grafts, allogenic and xenogenic bone or synthetic materials are generally applied (7). The use of various materials is well-documented for different indications of bone augmentation procedures (7).

A controversial subject is whether implant survival differs between augmented and non-augmented sites and if the time of augmentation and implant placement affects the treatment outcome (7,12-14). Implant failure is characterized by the loss of bone to implant contact, mobility, and presence of radiolucency around the implant surface (15). Implant survival depicts the permanence and the function of the implant in the oral cavity, whereas implant success demonstrates the lack of biological and technical complications as assessed by clinical and radiographic parameters (16). There is still lack of consensus regarding the effect of bone-related parameters on the risk of implant failure. The appropriate patient selection in implant dentistry is crucial and therefore identifying the reason of an implant failure is of paramount importance to prevent future implant loss.

The purpose of the present study was to retrospectively assess the failure rate of implants placed in augmented and non-augmented sites and to investigate whether the time of implant and bone placement are associated with the risk of implant failure in a university setting.

## Material and Methods

This study was approved by the Institutional Review Board of the University of Minnesota for medical record chart review (#1606M88402).

-Data extraction

Data were retrieved from the electronic database of the University of Minnesota School of Dentistry for implants placed and restored by dental students, postgraduate students and faculty for patients attending the university dental clinics between 2010 and 2016. The present investigation is a retrospective dental record-based study. Records of implants were deemed eligible for inclusion if they were placed in adult individuals, and all examined parameters were complete. All potentially eligible dental records were thoroughly examined and manually assessed for all parameters of interest.

-Treatment procedures

All bone augmentation procedures and surgical implant placements were performed at the University of Minnesota School of Dentistry by residents/postgraduate students under the direct supervision of a faculty member with advanced education training or performed by faculty members with specialty training. The augmentation techniques included guided bone regeneration, sinus augmentation and ridge preservation. In case of simultaneous implant placement, this was recorded and analyzed separately. The implant restoration was completed by dental students under direct supervision of a faculty member with advanced education training or performed by trained residents/postgraduate students or faculty. Various implant systems were utilized including Zimmer, Astra, Nobel, 3i, Straumann, and Biohorizons.

-Implant treatment outcome

Implant failure was defined as the removal of a dental implant for any reason including loss of integration, mobility, persistent pain, fracture and/or extensive bone loss as of the most recent follow-up appointment. Implant survival was recorded for any implant that was present in the oral cavity with the supporting restoration at the most recent recall appointment and exhibited no indication for implant explantation. The treatment outcome was included as a binary variable: implant failure/implant survival.

-Study variables

Datasheets were created using the electronic dental records of patients fulfilling the inclusion criteria. The following data were included:

• Patient’s age at the time of implant placement (in years)

• Patient’s gender (male/female)

• Tobacco use (yes/no)

• Implant location: jaw (maxilla/mandible) and region (anterior/posterior)

• Type of bone (native/augmented) 

• Sinus elevation procedure (yes/no)

• Ridge augmentation procedure (yes/no)

• Simultaneous sinus elevation and implant placement (yes/no)

• Simultaneous ridge augmentation and implant placement (yes/no)

• Multiple bone augmentation procedures (yes/no)

• Time to implant failure 

-Statistical analysis

The dependent variable in the analysis was the implant treatment outcome. Descriptive statistics including frequencies, means and standard deviations were calculated for all examined variables. Continuous variables were compared with the t-test, while categorical variables were expressed as proportions and compared with fisher’s exact test. Kaplan-Meier plots for the survival of both treatment modalities were created. Time to failure (date of procedure to date of visit with failure) was compared in Cox regression models between: a) implants placed in ridge augmented sites and implants placed in sites without ridge augmentation; b) implants placed in sinus augmented sites and implants placed in sites without sinus augmentation; c) implants placed simultaneously with ridge augmentation and implants placed without simultaneous ridge augmentation; d) implants placed simultaneously with sinus augmentation and implants placed without simultaneous sinus augmentation; as well as e) implants placed in sites with multiple grafting procedures and implants placed in sites without multiple grafting procedures. Patient-sites without a failure were censored at the last follow-up visit. Hazard ratios (HR) and their 95% confidence intervals (CI) are reported for each model. All tests of significance were evaluated at the 0.05 error level with a statistical software program (SPSS v.24.0, IBM, Armonk, NY, USA).

## Results

-Characteristics of the included studies 

A total of 4,645 dental records were identified in the electronic database of the University of Minnesota School of Dentistry and were screened for eligibility. Following the removal of duplicate and incomplete records, a total of 4,424 dental charts were further screened. A random selection of 553 implants were included in the analysis which represents the 1/8 (12.5%) of the available and eligible data. The characteristics of the included implants are shown in Table 1. Of the 553 implants, 278 (50.3%) were placed in males, while 69 (12.5%) of the sample were tobacco users. The mean age of the included population was 62.39±12.13 years. The location of the implants was 314 (56.8%) in the maxilla and 411 (74.3%) in the posterior regions. Bone augmentation was performed in 358 (64.7%) of the cases: sinus lift procedure (n=108, 19.5%) and ridge augmentation (n=250, 45.2%). Simultaneous ridge augmentation and implant placement were completed in 104 (18.8%) of the implants, whereas simultaneous sinus augmentation and implant placements was performed in 67 (12.1%) of the total implants. Multiple bone augmentation procedures were completed with 48 (8.7%) implants. Seventeen implants failed after a mean time of 6.29±6.75 months resulting in a 3.1% failure rate.

**Table 1:**
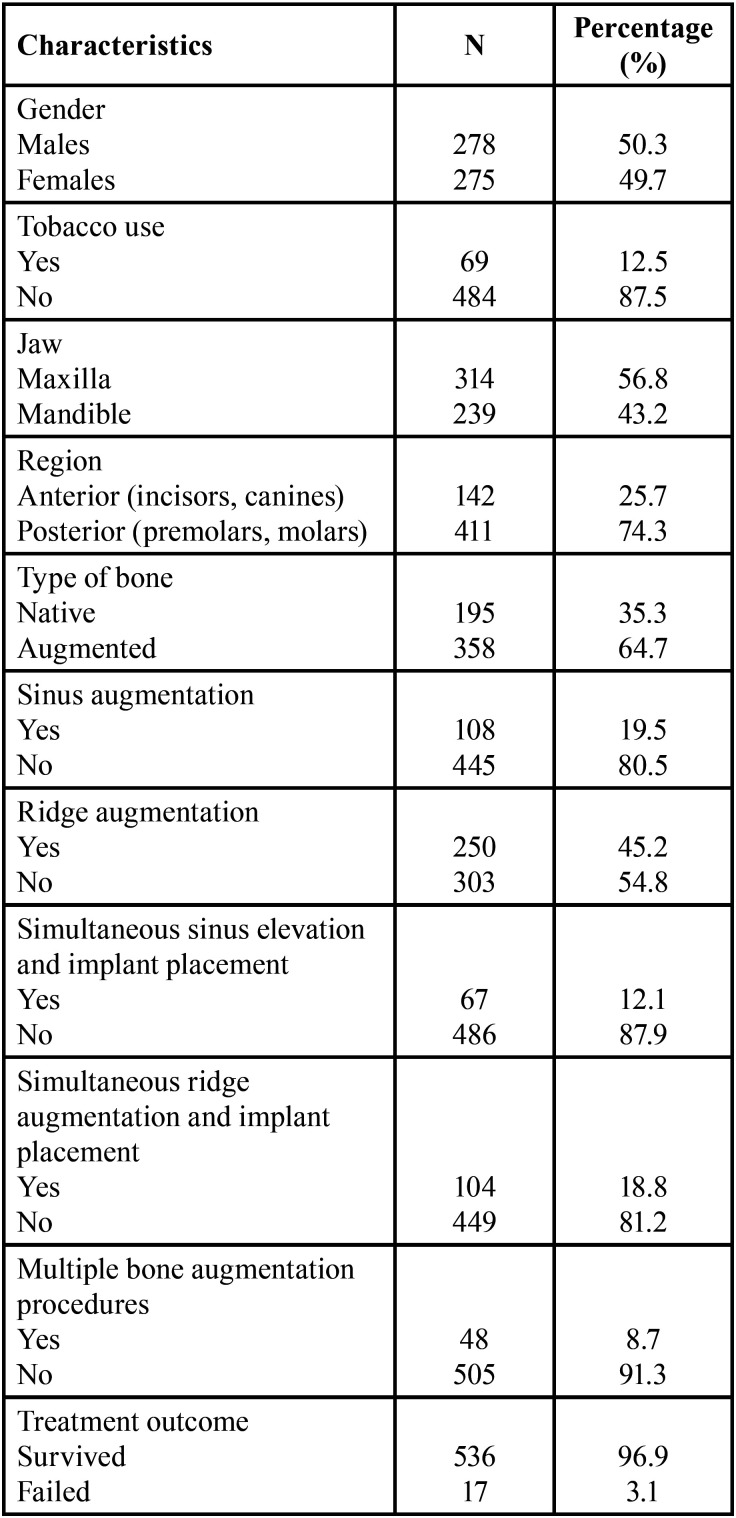
Characteristics of the included implants.

-Sinus augmentation

**Figure 1:**
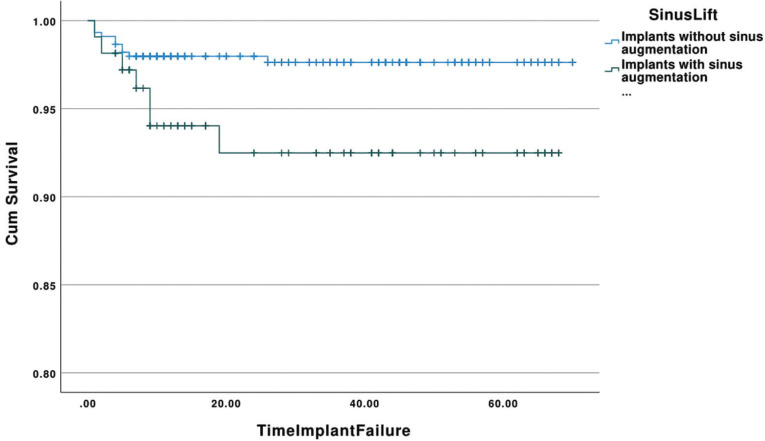
Kaplan-Meier curve showing the cumulative survival rates of implants placed in sinus grafted and non-grafted sinuses to time (in months).

The cumulative survival rates of implants placed in sinus grafted and non-grafted sinuses with respect to time (in months) is shown in Figure 1. The estimated mean survival time for implants placed in sinus grafted sites was 63.52 (95% Confidence Interval: 60.31-66.73) months with a range of 1-68 months, whereas implants placed in non-grafted sinuses showed a mean survival time of 68.49 months (95% Confidence Interval: 67.57-69.42) with a range of 1-70 months. The majority of the failed implants in the grafted group were removed within the first 9 months (85.7%). The overall survival rate for implants placed in sinus grafted sites was 93.5%, while implants inserted in non-grafted sinuses showed a survival rate of 97.8%. The survival rates between implants placed in sinus augmented and non-augmented sites were significantly different with implants placed in non-grafted sites demonstrating higher survival (*p*=0.018).

-Simultaneous sinus augmentation and implant placement

**Figure 2:**
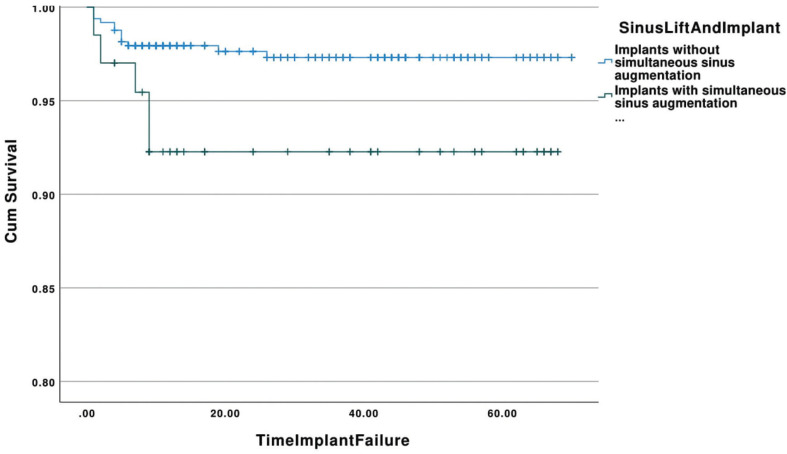
Kaplan-Meier curve showing the cumulative survival rates of implants placed simultaneously with sinus augmentation and implants placed with no simultaneous sinus augmentation with respect to time (in months).

The cumulative survival rates of implants placed simultaneously with sinus augmentation and implants placed with no simultaneous sinus augmentation with respect to time (in months) is demonstrated in Figure 2. The estimated mean survival time for implants placed simultaneously with sinus grafting was 63.18 (95% Confidence Interval: 59.12-67.24) months with a range of 1-68 months, whereas implants placed without simultaneous sinus grafting showed a mean survival time of 68.33 months (95% Confidence Interval: 67.39-69.26) with a range of 1-70 months. Most of the failed implants in the simultaneous sinus augmentation group were removed within the first 9 months (85.7%). The overall survival rate for implants placed simultaneously with sinus augmentation was 92.5%, while implants without simultaneous grafting in the sinuses showed a survival rate of 97.5%. The survival rates of implants placed simultaneously with sinus floor elevation and without simultaneous sinus grafting were significantly different with implants placed without simultaneous grafting demonstrating higher survival (*p*=0.025).

-Ridge augmentation

**Figure 3:**
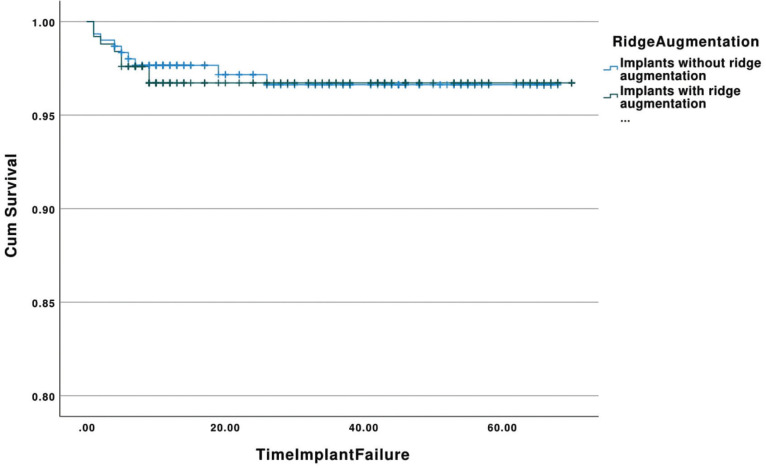
Kaplan-Meier curve showing the cumulative survival rates of implants placed in augmented alveolar ridges and non-grafted ridges with respect to time (in months).

The cumulative survival rates of implants placed in augmented alveolar ridges and non-grafted ridges with respect to time (in months) is shown in Figure 3. The estimated mean survival time for implants placed in ridge grafted sites was 67.86 (95% Confidence Interval: 66.39-69.32) months with a range of 1-70 months, whereas implants placed in non-grafted ridges showed a mean survival time of 66.03 months (95% Confidence Interval: 64.76-67.30) with a range of 1-68 months. The majority of the failed implants in the grafted group were removed within the first 5 months (75%). The overall survival rate for implants placed in ridge grafted sites was 96.8%, while implants inserted in non-grafted ridges showed a survival rate of 97.0%. No significant differences were found between the two groups (*p*=0.89).

-Simultaneous ridge augmentation and implant placement

**Figure 4:**
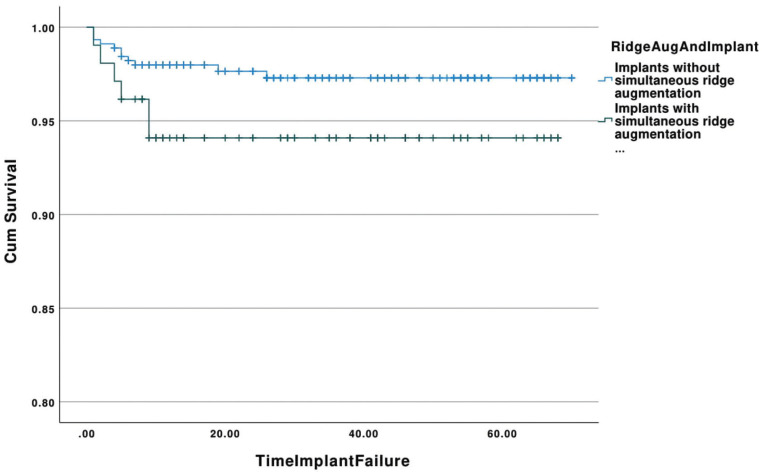
Kaplan-Meier curve showing the cumulative survival rates of implants placed simultaneously with ridge augmentation and implants placed with no simultaneous augmentation with respect to time (in months).

The cumulative survival rates of implants placed simultaneously with ridge augmentation and implants placed with no simultaneous ridge augmentation with respect to time (in months) is demonstrated in Figure 4. The estimated mean survival time for implants placed simultaneously with ridge grafting was 64.28 (95% Confidence Interval: 61.39-67.17) months with a range of 1-68 months, whereas implants placed in non-simultaneously grafted ridges showed a mean survival time of 68.33 months (95% Confidence Interval: 67.36-69.31) with a range of 1-70 months. All failed implants in the ridge augmentation group were removed within the first 9 months (100%). The overall survival rate for implants placed simultaneously with ridge augmentation was 94.2%, while implants placed in the control group showed a survival rate of 97.6%. Implants placed simultaneously with ridge augmentation showed similar survival with implants placed without simultaneous grafting (*p*=0.08).

-Multiple grafting

**Figure 5:**
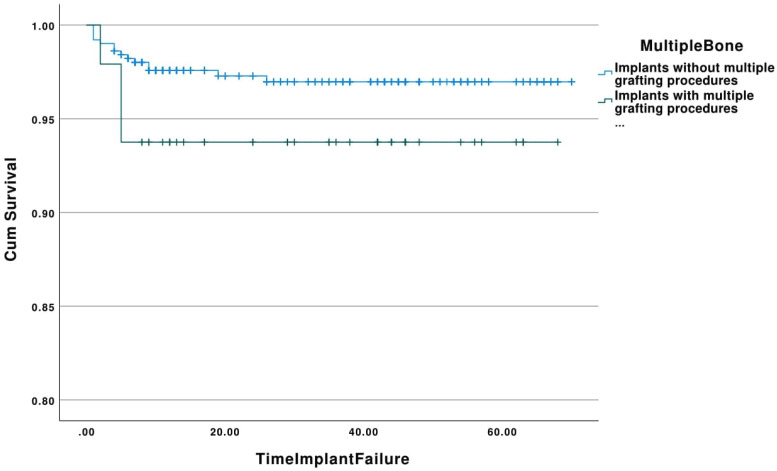
Kaplan-Meier curve showing the cumulative survival rates of implants placed in sites that had multiple grafting procedures and implants placed in sites with no multiple augmentation procedures with respect to time (in months).

The cumulative survival rates of implants placed in sites that had multiple grafting procedures and implants placed in sites with no multiple augmentation procedures with respect to time (in months) is shown in Figure 5. The estimated mean survival time for implants placed in sites that had multiple grafting procedures was 64.00 (95% Confidence Interval: 59.62-68.38) months with a range of 1-68 months, whereas implants placed in the control group showed a mean survival time of 68.12 months (95% Confidence Interval: 67.15-69.09) with a range of 1-70 months. All failed implants in the multiple grafting group were removed within the first 5 months (100%). The overall survival rate for implants placed in sites with multiple augmentation procedures was 93.8%, while implants placed in the control group showed a survival rate of 97.2%. This difference was not statistically significant (*p*=0.19).

-Multivariable Cox Regression

The multivariable Cox Regression model for implant failure: summary for the characteristics evaluated is shown in Table 2. A statistically significant association was found between tobacco use (*p*<0.001) and simultaneous ridge augmentation and implant placement (*p*=0.05) with the treatment outcome. Tobacco users were at 8.69 (Hazzard ratio: 8.69, 95% Confidence Interval: 2.91-25.94) significantly increased risk to experience implant failure than non-users (*p*<0.001). In addition, implants placed simultaneously with ridge augmentation exhibited a 4.72 (Hazzard ratio: 4.72, 95% Confidence Interval: 1.01-21.94) significantly higher risk to failure than those implants that did not have simultaneous ridge augmentation (*p*=0.05). All other examined parameters showed no significant association (*p*>0.05).

**Table 2:**
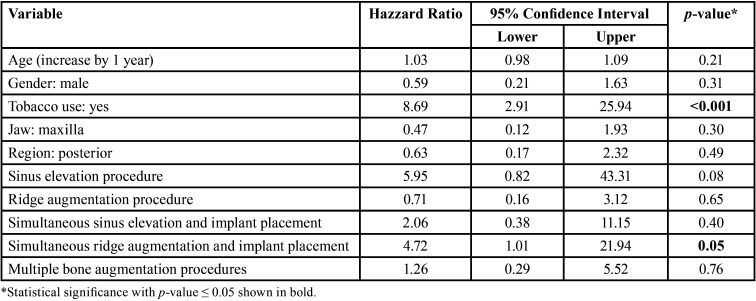
Multivariable Cox Regression Model for implant failure. Summary for the characteristics evaluated.

## Discussion

This retrospective electronic dental record-based investigation was undertaken to examine whether implants placed in grafted alveolar ridges and maxillary sinuses, with or without simultaneous grafting and implant placement, exhibit differences in implant survival long-term. Predicting the long-term treatment outcome following a surgical procedure has substantial importance in implant dentistry.

A total of 553 records of dental implants were included in the analysis and the incidence of implant failure in the present retrospective investigation was 3.1% which is similar to other studies’ reported findings (17-19). In addition, similar to other studies, tobacco users exhibited an 8.69 higher risk of implant failure than non-users (*p*<0.001). The effect of smoking on implant treatment has been highlighted in recent systematic reviews and it may be associated with the lower bone formation rate, the longer mineralization time as well as the abnormal angiogenesis that decreases the vascularization and the remodeling in smokers when compared to non-smokers (20-23). Studies with a similar design have identified smoking as a strong predictor of implant failure (17,18), while a meta-analysis of 292 studies showed that smoking leads to 140.2% higher risk for implant failure and 0.58 mm greater marginal bone loss (20).

The survival rate of implants placed in sinus grafted sites was 93.5% and it was significantly lower than implants placed in sites without sinus augmentation (*p*=0.018). In addition, when the implants were placed simultaneously with the sinus elevation, the rate dropped to 92.5% which was statistically significantly different than those implants placed without simultaneous sinus augmentation (*p*=0.025). Various studies have compared implant survival after sinus augmentation with implant survival in non-grafted sites. A systematic review of the literature compared the implant survival following sinus floor augmentation procedures with implants placed in pristine posterior maxillary bone and concluded that some of the included studies showed decreased survival rates following this type of procedure (12). In agreement with our findings, a prospective cohort study demonstrated that implants placed in augmented sinuses exhibited lower survival rates than implants placed in native bone and this difference was statistically significant (24). Del Fabbro and colleagues concluded that implant survival after sinus augmentation should be expected between 93.7% and 97.2% after at least 3 years, whereas Tong *et al*. showed that in a follow-up time between 6 to 60 months these rates are between 87% to 98% (25,26). Furthermore, short implants have been suggested as an alternative treatment option in order to avoid biological complications and higher failure rates associated with sinus augmentation (27).

On the contrary, other studies have shown that implants placed following maxillary sinus floor augmentation are highly predicTable and show similar survival rates with implants placed in non-grafted sinuses (28,29). Similar to our results, Cabezas-Mojon *et al*. have reported that the majority of implants failures are associated with implants inserted simultaneously with the graft procedure (14). In contrast with these reports, a meta-analysis that assessed the treatment outcome following simultaneous implant and sinus augmentation with delayed implant placement revealed no significant differences (30). These differences might be attributed to the residual bone height and the implant stability that can be achieved in such procedures (13,31,32).

With respect to the ridge augmentation procedures, in the present investigation the survival rate of implants placed in ridge augmented sites was 96.8% and the rate decreased to 94.2% when the implants had been placed simultaneously with the ridge augmentation procedure. No significant differences were identified when compared to non-augmented sites. Other published studies have shown similar results. No differences have been found for implants placed in native and augmented bone in a number of publications and a systematic review of the literature (33-36). The present retrospective investigation included implants inserted in both maxilla and mandible as well as anterior and posterior regions.

The multivariable Cox Regression model of the included data demonstrated that there is a statistically significant association between ridge augmentation and simultaneous implant placement with the implant treatment outcome (*p*=0.05). Ridge augmentation and simultaneous implant placement resulted in a 4.72 significantly higher risk of implant failure (*p*=0.05). Similar to our findings, Borba *et al*. adopted a generalized estimating equation statistical method to increase the reliability of the analysis and concluded that implants placed in augmented areas were more prone to failure and therefore bone grafting was a risk factor of implant failure (37). In agreement with these findings, Yang and colleagues after assessing the survival rates and risk factors of implants placed in an institution in China between 2006 and 2017 in the early stage showed that bone augmentation was a significant risk factor of implant failure (38). Furthermore, it has been suggested that alveolar ridges with severe bone loss should be augmented first and allow a sufficient recovery and healing time prior to implant placement to ensure adequate bone formation (39). In addition, it is expected that the marginal bone loss around implants inserted simultaneously with bone graft may be significantly higher than implants placed following the delayed protocol (40). Simultaneous implant placement and guided bone regeneration may exhibit limited benefits in severe atrophic ridges and it should be reconsidered especially when the clinicians aim to horizontally reconstruct more than 3 mm of bone (41).

The limitations of the present investigation are mainly associated with its retrospective design that may inherently lead to flaws. More specifically, the amount of the available alveolar bone height and width and the quantity of regenerative material used in the surgical procedures were not available. However, the effect of these possible factors may be minimized due to the standard of care which is followed in university dental clinics. In addition, the frequency and the duration of tobacco use was not recorded in the dental records and therefore could not be assessed. The variety of implant surgeons and implant systems used could also be considered a limitation of the study. However, all implant surgeons follow specific protocols that are adopted by the university and all implant systems used in university clinics must meet specific requirements. None of these parameters pose significant risk towards implant failure. Future randomized clinical trials should explore the effect of bone augmentation of the alveolar ridge and the maxillary sinus on implant survival either used simultaneously or not.

## Conclusions

Within the limitations of this retrospective randomly selected university-treated sample, implants placed in tobacco users as well as in augmented maxillary sinuses, simultaneously or in stages, pose a significantly higher risk of failure. Implants placed simultaneously with ridge augmentation also exhibited an increased risk of failure. Preventive measures should be taken to minimize the risk of implant loss.
